# An Angiotensin I Converting Enzyme Polymorphism Is Associated with Clinical Phenotype When Using Differentiation-Syndrome to Categorize Korean Bronchial Asthma Patients

**DOI:** 10.1093/ecam/nep053

**Published:** 2011-02-17

**Authors:** Sung-ki Jung, Jehyeon Ra, Jungchul Seo, Hee-Jae Jung, Jun-Yong Choi, Yong-Ju Cho, Mee-Suk Hong, Joo-Ho Chung, Jinju Kim

**Affiliations:** ^1^Division of Allergy and Respiratory System, Department of Oriental Internal Medicine, College of Oriental Medicine, Seoul 130-701, Republic of Korea; ^2^Department of Oriental Physiology and Kyung Hee East-West Pharmaceutical Research Institute, College of Pharmacy, Kyung Hee University, Seoul 130-701, Republic of Korea; ^3^Doo-Ree Korean Medicine Clinic, Jungang-dong, Gwacheon, Seoul, Republic of Korea; ^4^Kohwang Medical Research Institute, College of Medicine, Kyunghee University, Seoul, Republic of Korea

## Abstract

In this study, genetic analysis was conducted to investigate the association of angiotensin I converting enzyme (ACE) gene polymorphism with clinical phenotype based on differentiation-syndrome of bronchial asthma patients. Differentiation-syndrome is a traditional Korean medicine (TKM) theory in which patients are classified into a Deficiency Syndrome Group (DSG) and an Excess Syndrome Group (ESG) according to their symptomatic classification. For this study, 110 participants were evaluated by pulmonary function test. Among them, 39 patients were excluded because they refused genotyping. Of the remaining patients, 52 with DSG of asthma (DSGA) and 29 with ESG of asthma (ESGA), as determined by the differentiation-syndrome techniques were assessed by genetic analysis. ACE insertion/deletion (I/D) polymorphism analysis was conducted using polymerase chain reaction (PCR). Student's *t*, chi-square, Fisher and Hardy-Weinberg equilibrium tests were used to compare groups. No significant differences in pulmonary function were observed between DSGA and ESGA. The genotypic frequency of ACE I/D polymorphism was found to differ slightly between DSGA and ESGA (*P* = .0495). However, there were no significant differences in allelic frequency observed between DSGA and ESGA (*P* = .7006, OR = 1.1223). Interestingly, the allelic (*P* = .0043, OR = 3.4545) and genotypic (*P* = .0126) frequencies of the ACE I/D polymorphism in female patients differed significantly between DSGA and ESGA. Taken together, the results presented here indicate that the symptomatic classification of DSGA and ESGA by differentiation-syndrome in Korean asthma patients could be useful in evaluation of the pathogenesis of bronchial asthma.

## 1. Introduction

Asthma is a chronic inflammatory disease characterized by airway inflammation, increased mucus production, intermittent airway obstruction, and increased responsiveness [1–4]. The angiotensin I converting enzyme (ACE) inactivates bradykinin and tachykinins such as neurokinin A and substance P, which play important roles in asthma. Patients with asthma reportedly have bronchial hyper-reactivity to bradykinin when compared to control groups [5].

The ACE gene is located in the chromosome 17q23 region. Individual variability in the plasma ACE is associated with an insertion/deletion (I/D) polymorphism involving a 250-bp region situated in intron 16 of the ACE gene, which is known as the ACE I/D polymorphism [6]. According to the available medical literature, asthma patients in France are characterized by a higher prevalence of the ACE D/D genotype [7]. However, the distribution of the ACE genotype did not differ significantly between Turkish asthma patients and a control group [8]. Conversely, a significant association of the ACE I/D polymorphism with asthma was reported in asthma patients from the Czech Republic [9].

Traditional Korean medicine (TKM) uses a unique diagnostic technique known as differentiation-syndrome to analyze signs and symptoms of patients. In TKM, it is possible to have various treatments for the same disease because differentiation-syndromes are based on the overall analysis of symptoms and signs, causes, nature and the location of the illness. Wu and Guo [10] reported that chronic glomerulonephritis and differentiation-syndromes were related to cytochemical changes in the peripheral blood. In addition, Ji et al. [11] reported that 90 patients with chronic atrophic gastritis were divided into six groups by differentiation-syndrome. Wang et al. [12] reported the effects of planning treatments according to differentiation-syndrome.

Using differentiation-syndrome, asthma patients can be divided into two groups, the Deficiency Syndrome Group of Asthma (DSGA) and an Excess Syndrome Group of Asthma (ESGA). Deficiency refers to a deficiency of genuine Qi, lowered body resistance and declining function, including Yin and Yang-deficiency. Excess is characterized by the presence of excessive pathogenic factors that lead to an intense body reaction, or by the presence of pathological products due to the dysfunction of internal organs, such as stagnant Qi and blood, excessive fluid, retained phlegm and undigested food.

This study was conducted to determine if ACE I/D polymorphism was associated with a clinical phenotype of DSGA or ESGA in asthma patients. To accomplish this, we classified asthma patients into DSGA or ESGA categories and then analyzed the patients for the presence of ACE I/D polymorphism using polymerase chain reaction (PCR).

## 2. Methods

### 2.1. Subjects

One hundred and ten asthma patients participated in this study. All patients were diagnosed between December 2001 and September 2004 at the Kyung Hee University Oriental Medical Center. The diagnosis of asthma was confirmed based on a clinical history with current clinical symptoms such as episodic wheezing, chest tightness, dyspnea, and 15% or greater reversibility of forced expiratory volume at one second (FEV_1_) spontaneously or after treatment with a nebulized *β*_2_-agonist. Written informed consent was obtained from each subject and all experiments were conducted under protocols approved by the ethics review committee of the Oriental Medical Research Institute at the Kyung Hee University Medical Center.

### 2.2. Pulmonary Function Test

Pulmonary function was tested using a Vmax 22 D Sensormedix (Sensormedix Corporation, CA, USA) to measure the forced vital capacity (FVC), FEV_1_ and peak expiratory flow rate (PEF). Results are given as the percentage of the predicted values by Polgar and Morris.

### 2.3. Determination of ESGA or DSGA

All patients were diagnosed by TKM and then categorized into the DSGA or the ESGA. Patients were asked whether they had any of 37 symptoms relevant to the TKM diagnosis for asthma. These 37 symptoms were then arranged into seven categories of TKM patterns that each consisted of seven symptoms, with some symptoms being assigned to more than one category (Table 1) [13]. Each score of seven TKM was then calculated by counting the number of symptoms checked in each diagnostic category. Additionally, a score was determined for each of three typical symptoms (Table 1) [13] in each given diagnostic category. As a result, each diagnostic category comprised seven symptoms, of which three symptoms had 2 points and the other four symptoms had 1 point. Therefore, there was a possible total of 10 points for each diagnostic category. Any categories that received a score of 5 points or higher were considered to be relevant and the category with the highest score was chosen for the diagnosis of a given patient. If any categories had the same scores, an experienced TKM doctor chose the most relevant diagnosis based on a close examination of the patient. The seven diagnoses were then regrouped into two main categories, DSGA and ESGA. Specifically, individuals that showed patterns consistent with lung deficiency, heart-kidney deficiency and upper deficiency and lower excess were categorized as DSGA, whereas individuals categorized as having an external contraction of cold-wind pattern, phlegm-damp pattern, cold-phlegm pattern and phlegm-heat pattern were categorized as ESGA.

### 2.4. ACE Gene Polymorphism Analysis

**Figure 1:**
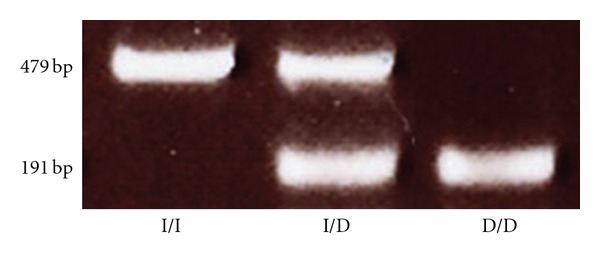
Electrophoresis of PCR products of the ACE gene. I; insertion, D; deletion, I/I; insertion homozygotes, I/D; insertion/deletion heterozygotes, D/D; deletion type homozygotes.

Peripheral blood samples were obtained from 81 subjects and collected in tubes containing ethylenediaminetetraacetic acid (EDTA). Genomic DNA was extracted using a DNA isolation kit for mammalian blood (Life Science & Biotech, Seoul, South Korea). The sense oligonucleotide primer used for PCR was 5′-CTG GAG ACC ACT CCC ATC CTT TCT-3′, and the antisense primer was 5′-GAT GTG GCC ATC ACA TTC GTC AGA T-3′ [14]. Briefly, 100 ng of template DNA were used for each PCR reaction, which was conducted under the following conditions: 35 cycles of 94°C for 45 s, 60°C for 50 s, 72°C for 1 min and a final extension at 72°C for 10 min. PCR products were electrophoresed on 2% agarose gels and stained with ethidium bromide to identify the following three possible patterns (Figure 1): I/I (a 479 bp fragment), D/D (a 191 bp fragment) and I/D (both 479 and 191 bp fragments) (NCBI: AF118569).

### 2.5. Statistical Analysis

All numerical variables are reported as the mean ± standard deviation. Analyses were conducted using SAS (Version 8.2. Cary, NC, USA). The differences in the asthma patient's ages and pulmonary functions were compared using a Student's *t*-test, while genotypic and allelic frequencies were compared using a chi-squared test. Chi-squared and Fisher tests were also used to determine if the observed genotype frequency was in Hardy-Weinberg equilibrium. A *P*-value of less than .05 was considered statistically significant for all analyses.

## 3. Results

### 3.1. Characteristics of Korean Asthma Patients

**Figure 2:**
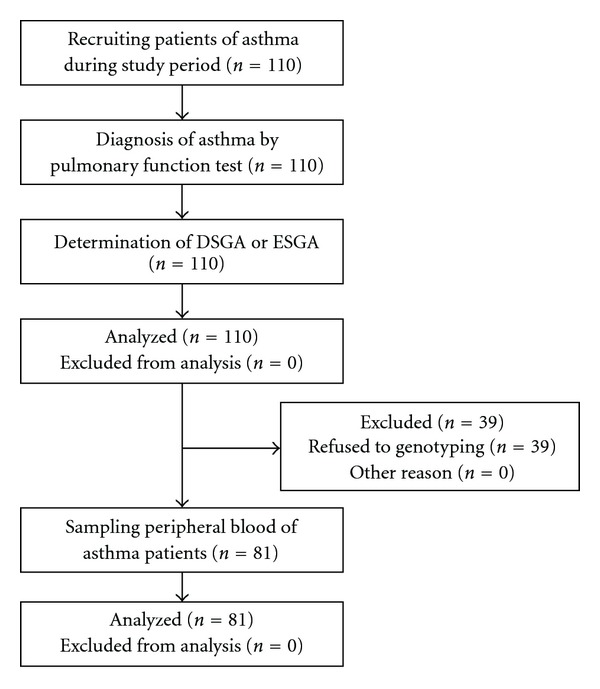
Diagram of study process.

All 110 patients had participated in this study. They had signed the informed consent form, tested pulmonary functions, and asked whether they had any of 37 symptoms relevant to TKM diagnosis for asthma. Of those 39 patients had refused genotyping analysis, therefore 81 patients were analyzed completely (Figure 2).

The characteristics of the 110 patients who were participated the pulmonary function test are presented in Table 2. They are composed of 49 male and 61 female, with a mean age of 49.9 ± 17.1 years. Of those, DSGA include 60 patients and ESGA was 50 patients. DSGA are composed of 31 male and 29 female, and ESGA are composed of 18 male and 32 female

### 3.2. Pulmonary Function Test in DSGA and ESGA

Pulmonary function test was performed in 110 asthma patients. Of the patients evaluated in this study, 60 were DSGA and 50 were ESGA. The mean FVC values were 87.6 ± 24.2, 86.2 ± 17.7 and 87.6 ± 20.2 for the entire group, DSGA patients and ESGA patients, respectively. The mean FEV_1_ levels were 77.5 ± 24.2, 76.0 ± 23.0, and 78.3 ± 26.5 for the entire group, DSGA patients and ESGA patients, respectively. The mean PEF values were 78.3 ± 28.8, 73.7 ± 25.4, and 79.1 ± 30.6 for all asthma patients, DSGA patients and ESGA patients, respectively. None of these values differed significantly between the DSGA and ESGA patients (Table 2).

### 3.3. Association between ACE I/D Polymorphism and Korean Asthma Patients

The observed genotype frequencies for the ACE I/D polymorphism in these Korean asthma patients are shown in Table 3. Of the 110 patients assessed using the pulmonary function test, 39 were excluded from subsequent evaluation because they refused to undergo genetic analysis. Therefore, 81 patients were evaluated for the presence of the ACE I/D polymorphism by PCR.

The allelic frequencies of ACE did not differ significantly between the DSGA and ESGA groups (*P* = .7006, OR = 1.1223). However, the genotypic frequency of ACE differed significantly between the ESGA and DSGA groups (*P* = .0445) (Table 3). The allelic and genotypic frequencies of ACE did not differ between male and female patients (*P* = .9907 and *P* = .9605, resp.). In addition, there were no significant differences in the allelic and genotypic frequencies of ACE of males in the ESGA and DSGA groups (*P* = .8051 and *P* = .5795, resp.). However, the allelic and the genotypic frequencies of the ACE I/D polymorphism differed significantly between DSGA and ESGA females (*P* = .0043, OR = 3.4545 and *P* = .0126, resp.) (Table 4).

## 4. Discussion and Conclusion

Bronchial asthma is influenced by many environmental and genetic factors [15]. One of the genetic factors believed to play an important role in asthma is the ACE I/D polymorphism [16]. In the present study, bronchial asthma patients were classified into the DSGA or ESGA group based on their differentiation-syndrome, which is a diagnostic method based on TKM theory. Deficiency syndrome is characterized by a deficiency of genuine Qi, lowered body resistance and declined function, including Yin and Yang deficiency. Excess syndrome is characterized by stagnant Qi, raised body resistance and excessive function, including Yin and Yang excessiveness. DSGA includes patients with patterns of lung deficiency, heart-kidney deficiency, and upper deficiency and lower excess deficiency. Conversely, ESGA patients are characterized by patterns of external contraction of cold-wind, phlegm-damp patterns, cold-phlegm patterns and phlegm-heat patterns (Table 1) [13]. Through diagnosis of DSGA and ESGA, a TKM doctor can prescribe various treatments for bronchial asthma.

DSGA and ESGA have been extensively studied in Korea. Choi et al. [13] analyzed the therapeutic effects of treatments prescribed based on criteria for Defiency-Excess Syndromes of asthma. In addition, other studies have been conducted to evaluate the effects of Gamipaimo-tang on asthma associated with excess syndrome [17], and the effect of Chuongsangboha-tang on asthma associated with deficiency syndrome [18]. Furthermore, Yu et al. [19] reported an association between the glutathione-s-transferase M1/T1 gene polymorphism and Deficiency-Excess differentiation.

In the present study, the clinical phenotypes of DSGA and ESGA were not associated with the pulmonary function test. Furthermore, no differences between DSGA and ESGA patients were observed when the asthmatic patients were categorized according to their distribution of ACE allelic frequencies. Consequently, based on the results of the current study, DSGA and ESGA do not appear to be related to the degree of airway obstruction. A high prevalence of ACE deletion polymorphisms were found in DSGA patients (frequency of D > I allele in ESGA and DSGA, and the distribution rate of DD > ID > II genotype in DSGA) (Table 3). In addition, the ACE allelic and genotypic frequencies were found to differ significantly between females in the DSGA and ESGA groups.

Many other researchers and practitioners of complementary and alternative medicine including TKM doctors understand that all-inclusive diagnostic approach is essential for new perspectives and ideas to this developmental endeavor [20].

In conclusion, the results of this study suggest that DSGA and ESGA are significant clinical phenotypes associated with the ACE polymorphisms in Korean asthma patients. In addition, these findings may provide a new approach to investigation of the etiology of asthma.

## Figures and Tables

**Table 1:** Summary of criteria for DSGA and ESGA [13].

DSGA
Lung deficiency pattern
Paroxysmal cough, frequent breathing, weakness in voice^a^
Undertone and weakness in the sound of cough^a^
Sweating in daytime^a^, dryness of mouth, mild face flush
Heart-kidney deficiency pattern
Asthmatic symptoms for more than 5 years, difficulty and
shortness of breath in inspiration^a^
Difficulty in breathing on movement^a^, frequent fatigue,
swelling tendency and/or frequent urination,
Night sweats^a^, cold hands and feet
Upper excess and lower deficiency pattern
Paroxysmal cough, excessive sputum^a^, difficulty in breathing
on movement^a^
Lumbago and/or cold low back^a^, cold hands and feet
Swelling tendency and/or frequent urination, palpitations
ESGA
External contraction of cold-wind pattern
Paroxysmal cough, watery sputum, white sputum, chillness^a^
Headache^a^, generalized pain^a^, no thirst
Phlegm-damp pattern
Paroxysmal cough, excessive sputum, viscous sputum^a^
Unpleasantness after expectoration, chest discomfort
Nausea^a^, loss or reduction in appetite^a^
Cold-phlegm pattern
Exacerbation by cold weather or cold wind, watery sputum
White sputum^a^, foamy sputum, chest discomfort
Dark and bluish tone of face^a^, feeling of cold body and
preference for drinking warm water^a^
Phlegm-heat pattern
Frequent breathing^a^, high tone and coarseness in the sound
of cough
Viscous sputum, yellowish sputum^a^, chest discomfort
Flushed face with sweating, thirst for water^a^

DSGA, Deficiency Syndrome Group of Asthma; ESGA, Excess Syndrome Group of Asthma.

^a^Important symptoms for each pattern.

**Table 2:** Clinical characteristics of the 110 Korean asthma patients and distribution of pulmonary functions in DSGA and ESGA.

	Asthma patients (*n* = 110)	DSGA (*n* = 60)	ESGA (*n* = 50)	*P*-value
Age (years)	49.9 ± 17.1	50.0 ± 16.2	49.9 ± 18.3	NS^a^
Sex (male/female)	49/61	31/29	18/32	NS^b^
Pulmonary functions				
FVC predicted (%)	87.6 ± 24.2	86.2 ± 17.7	87.6 ± 20.2	NS^a^
FEV_1_ predicted (%)	77.5 ± 24.2	76.0 ± 23.0	78.3 ± 26.5	NS^a^
PEF predicted (%)	78.3 ± 28.8	73.7 ± 25.4	79.1 ± 30.6	NS^a^

Values are mean ± SD; DSGA, Deficiency Syndrome Group of Asthma; ESGA, Excess Syndrome Group of Asthma; FVC, forced vital capacity; FEV_1_, forced expiratory volume at 1 s; PEF, peak expiratory flow rate; NS, no significance.

^a^Student's *t*-test.

^b^Chi-squared test.

**Table 3:** Distribution of allelic and genotypic frequency for ACE I/D polymorphism in Korean populations.

		Allele frequency (%)		Genotype frequency (%)			HWE
	*N*	D	I	D/D	I/D	I/I	*P*-value
DSGA	52	67 (64.42)	37 (35.58)	23 (44.23)	21 (40.38)	8 (15.38)	.3908
ESGA	29	63 (67.02)	31 (32.98)	5 (17.24)	17 (58.62)	7 (24.14)	.3377
*P*-value		0.7006^a^		0.0445^b^			
Odds ratio (95% CI)		1.1223 (0.6232 − 2.0211)					

DSGA, Deficiency Syndrome Group of Asthma; ESGA, Excess Syndrome Group of Asthma; HWE, Hardy-Weinberg equation.

^a^Chi-squared test with 2 × 2 contingency table.

^b^Fisher's test with 3 × 2 contingency table.

**Table 4:** Comparison of the frequency of ACE I/D polymorphism between male and female DSGA and ESGA patients.

		Allele frequency (%)		Odds ratio	*P*-value	
	*N*	D	I	(95% CI)		
Male	33	39 (59.00)	27 (40.91)	0.9962	.9907^a^	
Female	48	59 (59.00)	41 (41.00)	(0.5295–1.8745)		
Male						
DSGA	19	23 (60.53)	15 (39.47)	1.15	.8051^b^	
ESGA	14	16 (57.14)	12 (42.86)	(0.4266–3.1000)		
Female						
DSGA	33	44 (66.67)	22 (33.33)	3.4545	.0043^b^	
ESGA	15	11 (36.67)	19 (63.33)	(1.4019–8.5125)		

		Genotype frequency (%)			*P*-value	HWE
	*N*	D/D	I/D	I/I		*P*-value

Male	33	12 (36.36)	15 (45.45)	6 (18.18)	.9605^c^	.7311
Female	48	16 (33.33)	23 (47.92)	9 (18.75)		.8852
Male						
DSGA	19	8 (42.11)	7 (36.84)	6 (18.18)	.5795^d^	.3182
ESGA	14	4 (28.57)	8 (57.14)	2 (14.29)		.5329
Female						
DSGA	33	15 (45.45)	14 (42.42)	4 (12.12)	.0126^d^	.794
ESAG	15	1 (6.67)	9 (60.00)	5 (33.33)		.2583

DSGA, Deficiency Syndrome Group of Asthma; ESG, Excess Syndrome Group of Asthma; HWE, Hardy-Weinberg equation.

^a^Male versus female using the chi-squared test with a 2 × 2 contingency table.

^b^DSGA versus ESGA sex distinction using the Fisher's test with 2 × 2 contingency table.

^c^Male versus female using the chi-squared test with 3 × 2 contingency table.

^d^DSGA versus ESGA sex distinction using the Fisher test with 3 × 2 contingency table.
